# Rapidly Growing Malignant Peripheral Nerve Sheath Tumors Arising From Neurofibromatosis Type 1: A Case Report by Rehabilitation Physicians

**DOI:** 10.7759/cureus.79995

**Published:** 2025-03-03

**Authors:** Koji Hayashi, Yuka Nakaya, Toyoaki Miura, Asuka Suzuki, Hiroaki Maeda, Mamiko Sato, Yasutaka Kobayashi

**Affiliations:** 1 Department of Rehabilitation Medicine, Fukui General Hospital, Fukui, JPN; 2 Graduate School of Health Science, Fukui Health Science University, Fukui, JPN

**Keywords:** malignant peripheral nerve sheath tumor (mpnst), malignant spinal tumor, nerve schwannoma, neurofibromatosis type 1 (nf-1), spinal cord tumor

## Abstract

We describe a case of a 40-year-old Japanese woman with rapidly growing malignant peripheral nerve sheath tumors (MPNSTs) arising from neurofibromatosis type 1 (NF-1).

The patient presented numbness in both legs, back pain, and gait disturbances. Magnetic resonance imaging (MRI) revealed a spinal tumor at the thoracic level. To resolve her symptoms, a laminectomy and intradural tumor resection were performed. The tumor was diagnosed as a neurofibroma with no malignant characteristics. After the surgery, she participated in a rehabilitation program aimed at promoting independence in daily activities and enhancing muscle strength. Initially, her walking ability showed improvement; however, she soon experienced complications, including challenges with bowel movements and a gradual decline in her walking function. A follow-up MRI on the 67th day post-surgery showed tumor regrowth that necessitated reoperation. After the surgery, the neurological symptoms improved temporarily, but they worsened again, ultimately leading to a shift to palliative care and her demise several days later.

This case underscores the challenges in pathological diagnosis and the aggressive nature of MPNSTs, emphasizing the need for vigilant monitoring and timely intervention. Despite initial surgical success, rapid tumor growth can occur during rehabilitation, highlighting the importance of a multidisciplinary approach for accurate diagnosis and treatment. Early detection of tumor progression through meticulous neurological monitoring and prompt surgical consultation are critical for optimal outcomes. Further research into more definitive diagnostic tools and effective treatment strategies for MPNSTs is crucial to improve patient care.

## Introduction

Malignant peripheral nerve sheath tumors (MPNSTs) are uncommon and aggressive soft tissue sarcomas that originate from the nerve sheath, representing about 5-10% of all soft tissue sarcomas [1-3]. The overall lifetime incidence in the general population is estimated to be 0.001% (1 in 100,000) [1]. However, individuals with neurofibromatosis type 1 (NF1) face a significantly increased lifetime risk, estimated at around 10% [1]. Notably, up to 50% of all cases of MPNSTs occur in patients with NF1, underscoring the critical importance of early diagnosis and management for this population [1,4]. The World Health Organization (WHO) first classified MPNSTs as a soft-tissue sarcoma in 2013, identifying subtypes such as epithelioid MPNSTs, malignant triton tumor, and glandular MPNSTs [5]. While MPNSTs predominantly originate from Schwann cells or pluripotent cells of neural crest origin [6], their precise cellular makeup can be intricate, incorporating various tissue types that warrant further exploration [5].

MPNSTs generally arise between the ages of 30 and 50, although in NF1 patients, the average age of onset is typically 10 years younger [4]. Although MPNSTs primarily occur in the proximal limbs, they can also manifest in the trunk, head, and neck [4]. Common clinical symptoms encompass pain and numbness, yet these manifestations are nonspecific and can complicate the differentiation from other nerve lesions [4,7-10]. Because of their aggressive growth and tendency to spread, MPNSTs carry a poor prognosis and are the primary cause of adult mortality associated with NF1 [11]. Similar to other soft tissue sarcomas, effective treatment for MPNSTs requires complete surgical resection with adequate margins, which is often followed by radiation therapy and/or chemotherapy [11]. Although complete surgical resection with wide negative margins is still the only established curative approach for localized disease, the ability to perform resection depends on factors such as the location and size of the tumor, highlighting the importance of prompt and early diagnosis. Diagnosing and treating MPNSTs remains a significant challenge, and the prognosis is generally poor, with a high associated mortality rate [12]. Previous research has indicated that the overall five-year survival rate ranges from 50% to 60%, with a median survival time of six years for patients with MPNSTs [13].

This report presents a case of MPNSTs in which the patient experienced rapid tumor growth twice during the rehabilitation course immediately following tumor resection, ultimately resulting in death.

## Case presentation

**Figure 1:**
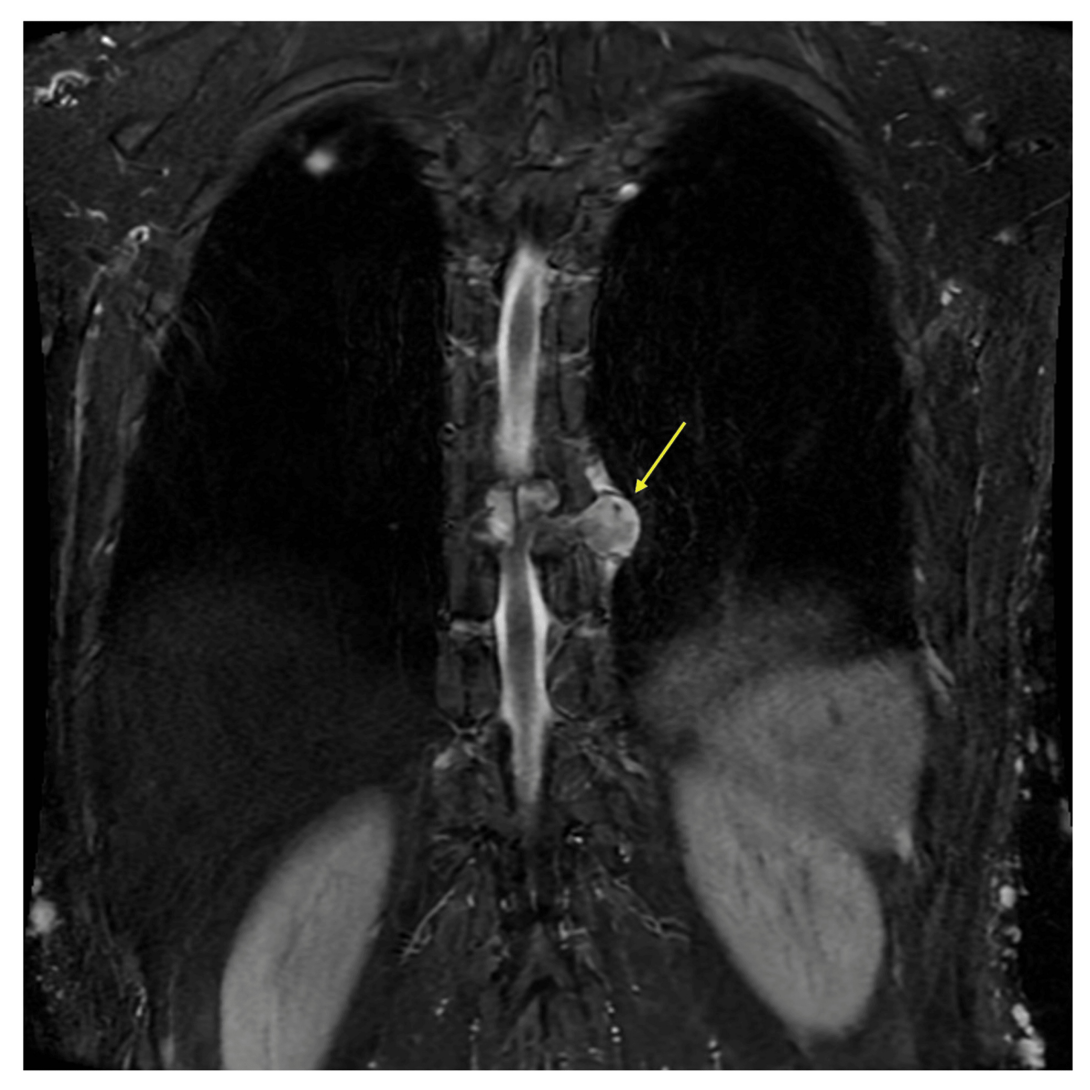
Thoracic magnetic resonance imaging on admission at the previous hospital. T2-weighted thoracic spine MRI revealed a spinal tumor located in the left thoracic spinal cord at the Th9-10 level (arrow).

A 40-year-old Japanese woman developed numbness in both legs, back pain, and gait disturbances after experiencing these symptoms for a month and visited her previous hospital. She had a prior diagnosis of NF1. Magnetic resonance imaging (MRI) revealed a spinal tumor located in the left thoracic spinal cord at the Th9-10 level (Figure 1), prompting her to undergo a laminectomy at Th8-9, intradural tumor resection, and partial resection of the epidural tumor. Pathological evaluation showed no malignant findings, and the tumor was diagnosed as a neurofibroma. On the 37th day after surgery, the patient was referred to our hospital for rehabilitation therapy.

**Figure 2:**
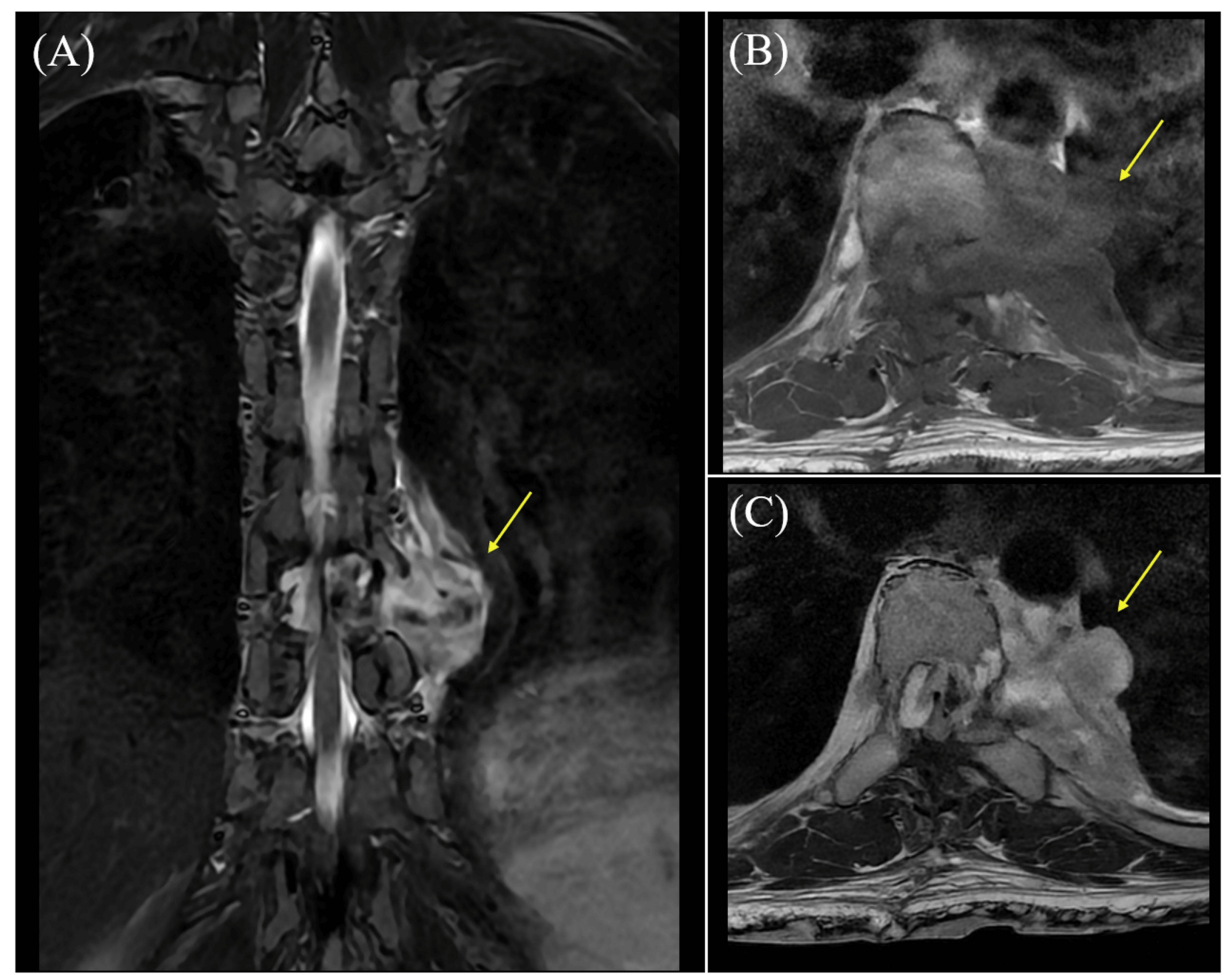
Follow-up MRI on day 70 after the initial surgery. (A) Thoracic spine MRI using T2-weighted imaging (coronal section) revealed a mass measuring 63 x 55 mm extending from the Th9 vertebral body, arch, and epidural space to the left posterior mediastinum through the left foramina of Th9/10 (arrow). (B) T1-weighted thoracic spine MRI revealed a lobulated mass with hypointensity (arrow). (C) T2-weighted thoracic spine MRI showed a lobulated mass with hypointensity, accompanied by uneven abnormal signals within the mass (arrow).

She had multiple raised skin lesions all over her body, including her face. Neurological examination revealed muscle weakness at the Medical Research Council (MRC) grade 4 level in the bilateral iliopsoas, left quadriceps, bilateral hamstrings, and left anterior tibialis. There was increased tendon reflex activity in both lower limbs, a positive right Babinski sign, positive right foot clonus, and paresthesia in both lower limbs. No cranial nerve deficits, upper limb paralysis, or ataxia were noted. Although the patient experienced some difficulty with urination, they were able to defecate independently. She used a walker for mobility due to lower limb weakness. We initiated rehabilitation therapy with the short-term goals of promoting independence in basic activities of daily living (ADLs), improving muscle strength and range of motion, and long-term goals of returning to daily life, providing psychological support and encouraging social participation, and enhancing the quality of life. Specifically, we planned to implement muscle strengthening exercises, range of motion exercises and stretching, balance training and exercises for fall prevention, gait training, and instruction in ADLs (bathing, dressing, eating, etc.). These rehabilitative efforts led to some improvement in the patient's walking function. She was able to walk independently for short distances and train with a walking stick for longer distances. However, over a period of 15 days, starting on the 46th day after surgery, she tried various laxatives but was unable to have a bowel movement. On the 67th day after surgery, walking deteriorated, and the patient was no longer able to walk using a walker. Thoracic spine MRI revealed a 63 x 55 mm sized, lobulated mass extending from the Th9 vertebral body, arch, and epidural space to the left posterior mediastinum through the left foramina of Th9/10. This mass demonstrated hypointensity on T1-weighted images and hyperintensity on T2-weighted images with internal heterogeneity (Figure 2). On the 75th day after surgery, she was transferred back to her previous hospital.

**Figure 3:**
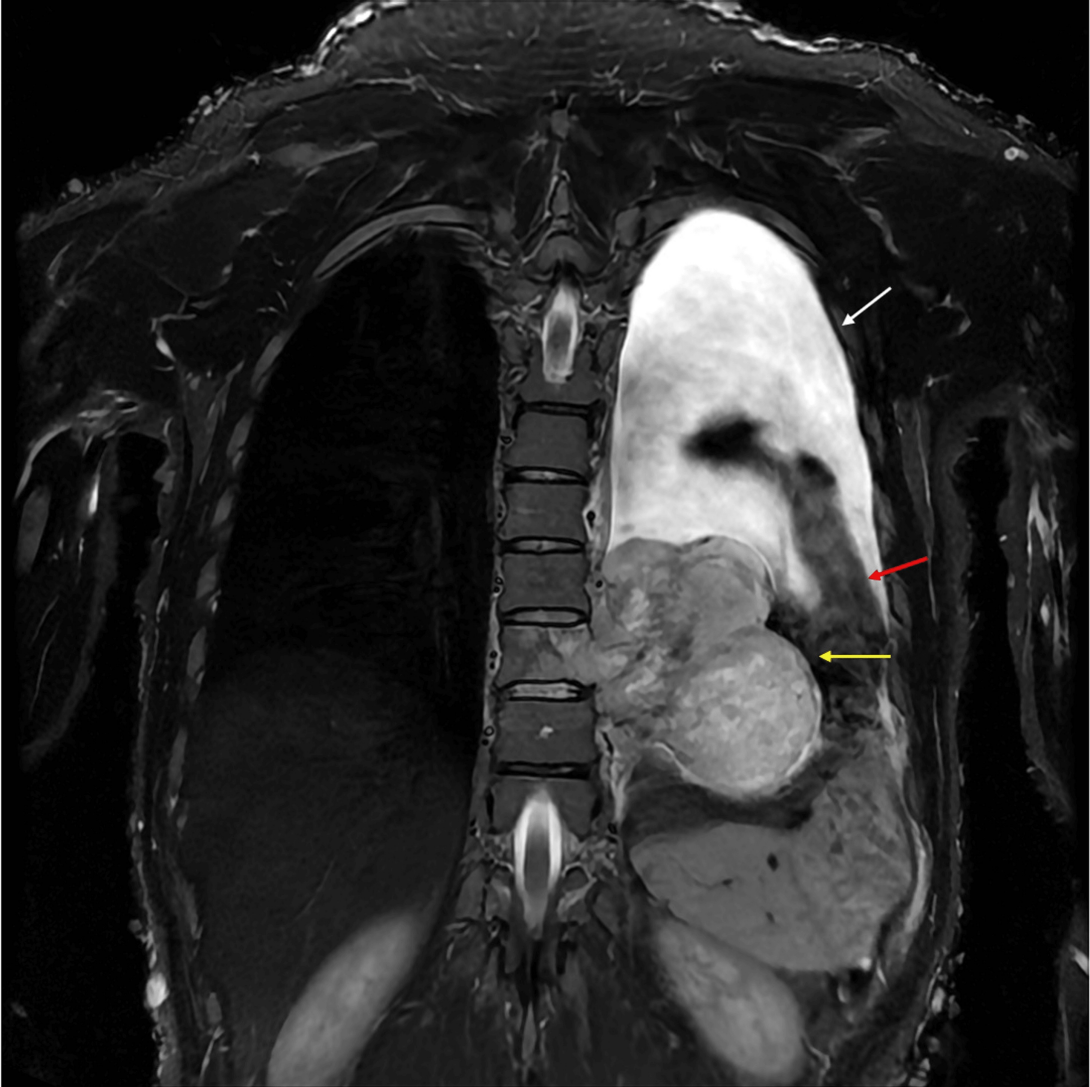
Follow-up thoracic spine MRI on the 126th day. The MRI revealed significant tumor enlargement (diameter 98 mm) with lobulation (yellow arrow). Pleural effusion (white arrow) and collapsed lungs (red arrow) were also noted. Additionally, the skin surface exhibited numerous tumors associated with neurofibromatosis type 1.

On the 80th day after her initial surgery, she underwent a second surgery for spinal tumor removal. The tumor, which had progressed from the epidural space into the Th9 vertebral body, was resected. Pathologists deemed no malignant findings, and the tumor was diagnosed as a neurofibroma. A postoperative MRI performed at the previous hospital confirmed that spinal cord compression had been alleviated. As a result of postoperative rehabilitation at her previous hospital, she regained walking with a walker. On the 113th day after the initial surgery, the patient was transferred back to our hospital for rehabilitation. Neurological examination revealed spasticity in both lower limbs with the following MRC grades (right/left): iliopsoas 3/2; gluteus maximus 2/2; quadriceps 4/4; tibialis anterior 3/2; gastrocnemius 3/2; left-dominant hypoesthesia in both lower limbs; and hyperreflexia in both lower limbs. In terms of ADLs, the patient was independent in getting in and out of bed and transferring with the use of handrails. While they could use a walker, they exhibited instability, requiring supervision. However, after transferring to our hospital, the patient's back pain, lower limb numbness, and paralysis in both legs worsened progressively. She and her family sought a second opinion and visited another advanced medical center on the 125th day after the initial surgery, where she was diagnosed with MPNSTs. The thoracic spine MRI on the 126th day showed significant tumor enlargement (diameter 98 mm) (Figure 3). By the 127th day after the initial surgery, both legs had reached MRC grade 1. On the 128th day, she returned to her previous hospital; however, surgery was deemed impossible, and the focus shifted to palliative care, leading to her death several days after readmission.

## Discussion

We describe a case of an aggressively growing MPNST in a patient undergoing rehabilitation immediately following surgery for NF-1. Although she was ultimately diagnosed with an MPNST, her surgeon initially referred her to us, the rehabilitation physicians, under the impression that she had benign neurofibromatosis. The tumor experienced two episodes of rapid growth; the first instance was successfully surgically removed, but in the second instance, surgery was not feasible, and the patient received palliative care. After both episodes of tumor growth, there was a slight improvement in neurological symptoms following surgery; however, new neurological deficits subsequently emerged, and imaging studies confirmed rapid tumor growth. Ultimately, she was transferred back to the original hospital after the second episode of tumor enlargement, where she succumbed to the disease.

The mechanisms underlying the development of MPNSTs from NF1 are estimated to involve two key pathways: genetic mechanisms, as well as signaling pathways and the microenvironment [13]. Recent advancements in genetic sequencing have revealed that the progression from NF1 to MPNSTs involves multiple genetic alterations, particularly affecting Schwann cells [13]. The loss of the NF1 gene is a significant initiating factor, but it is not sufficient on its own for malignant transformation, suggesting that additional genetic changes are required. Notably, the deletion of CDKN2A is commonly observed in MPNST cases, promoting the tumor's premalignant characteristics. Additional mutations, such as those in TP53, EGFR, and PTEN, have been identified, revealing potential independent pathways leading to MPNST development. Sporadic MPNSTs may involve different mutations, such as BRAF and NRAS, which are not typically seen in NF1-associated tumors [13].

On the other hand, the RAS signaling pathway becomes aberrantly activated due to alterations in the NF1 gene, leading to increased cell proliferation through downstream pathways like RAF-MEK-ERK and PI3K-AKT-mTOR [13]. mTOR signaling, in particular, has been linked to tumor growth and may serve as a therapeutic target, although clinical effectiveness has varied. The tumor microenvironment also plays a crucial role, with differences in immune cell populations noted in NF1 heterozygous conditions that may enhance tumor growth. These factors contribute to the complex pathogenesis of MPNSTs [1].

In summary, the transition from NF1 to MPNSTs is influenced by multiple genetic mutations and disrupted signaling pathways, alongside an altered tumor microenvironment, highlighting the complexity of its pathogenesis.

At present, there are no precise diagnostic criteria for MPNSTs, which complicates the differentiation from other soft tissue tumors and peripheral nerve sheath tumors [13]. MRI is crucial for the preoperative diagnosis of MPNSTs, with several key characteristics that can aid in differentiating MPNST from benign peripheral nerve sheath tumors such as NF1 [14]. These distinguishing features include a tumor size greater than 5 cm accompanied by ill-defined margins, the presence of peritumoral edema-particularly when the tumor is superficially located-intratumoral lobulation, an absence of the target sign, and evidence of bone destruction, which indicates malignancy. It is important to note that no single feature is sufficient for a definitive diagnosis; thus, utilizing a combination of two or more of these characteristics is advantageous for early detection and improving prognosis, especially in patients with NF1 [14].

Additionally, positron emission tomography/computed tomography (PET/CT) is recommended for diagnosing MPNSTs, as it typically shows elevated uptake of 18F-FDG. While the optimal PET/CT threshold for distinguishing MPNSTs from benign tumors associated with NF1 is still debated, some studies suggest that 18F-FDG PET/CT provides sufficient accuracy for detecting MPNSTs [15].

MPNST diagnosis is challenging due to the lack of definitive markers, especially in sporadic cases [13]. Diagnosis relies on excluding other soft tissue sarcomas, as MPNSTs are nerve-derived tumors often attached to nerve trunks. Microscopically, MPNSTs display diverse morphologies, with elongated, pointed nuclei and limited cytoplasm. Common features include cell necrosis, mitosis, and hemorrhage [13]. While these findings are not specific, they aid in differentiating MPNSTs from other tumors [13].

In our case, because the tumor originated in the thoracic spinal cord, it was challenging to achieve a large negative margin during surgery. Additionally, the pathologists consistently classified the tumor as benign, and the malignant findings were not communicated to the patient or his family, nor to the rehabilitation physicians. However, radiographic features, including aggressive tumor enlargement at the resection site, intratumoral lobulation, absence of a target sign, and size greater than 5 cm, were consistent with MPNSTs. Rehabilitation physicians should closely monitor neurological improvement daily, as the transformation of NF1-related tumors into MPNSTs can occur suddenly and rapidly. It is crucial to remember that MPNSTs can grow aggressively, potentially leading to irreversible deterioration even during the rehabilitation period within months of surgery. Although we were unable to perform this in our facility due to lack of PET-CT, it may be worth considering if malignant findings are suspected. In addition, since the diagnosis of MPNSTs based on pathological findings can sometimes be difficult, evaluation should be performed in conjunction with other modalities. If any signs of deterioration arise, immediate imaging tests and consultation with a surgeon are essential to facilitate early and appropriate treatment planning.

## Conclusions

This case highlights the challenges of managing MPNSTs, particularly the potential for rapid tumor growth even during the rehabilitation period. While pathologic diagnosis can be complex, a multidisciplinary approach utilizing imaging modalities such as MRI and PET-CT is essential for accurate assessment. Rehabilitation physicians play a crucial role in detecting early signs of tumor progression by diligently monitoring neurological function. Prompt consultation with a surgeon is critical when suspicion arises, and every effort should be made to achieve a wide surgical margin, despite potential location-specific challenges. This case underscores the importance of vigilance and timely intervention to improve outcomes for patients with MPNSTs. Further research is warranted to develop more definitive diagnostic tools and effective treatment strategies for this aggressive tumor type.
